# Accuracy Evaluation of Videogrammetry Using A Low-Cost Spherical Camera for Narrow Architectural Heritage: An Observational Study with Variable Baselines and Blur Filters

**DOI:** 10.3390/s19030496

**Published:** 2019-01-25

**Authors:** Zheng Sun, Yingying Zhang

**Affiliations:** 1 School of Architecture, Nanjing Tech University, Nanjing 211800, China; 2 School of Architecture, Southeast University, Nanjing 210096, China; 230139003@seu.edu.cn

**Keywords:** spherical camera, videogrammetry, baseline, narrow architectural heritage, 3D reconstruction, point cloud

## Abstract

Three-dimensional (3D) reconstruction using video frames extracted from spherical cameras introduces an innovative measurement method in narrow scenes of architectural heritage, but the accuracy of 3D models and their correlations with frame extraction ratios and blur filters are yet to be evaluated. This article addresses these issues for two narrow scenes of architectural heritage that are distinctive in layout, surface material, and lighting conditions. The videos captured with a hand-held spherical camera (30 frames per second) are extracted to frames with various ratios starting from 10 and increasing every 10 frames (10, 20, …, n). Two different blur assessment methods are employed for comparative analyses. Ground truth models obtained from terrestrial laser scanning and photogrammetry are employed for assessing the accuracy of 3D models from different groups. The results show that the relative accuracy (median absolute errors/object dimensions) of spherical-camera videogrammetry range from 1/500 to 1/2000, catering to the surveying and mapping of architectural heritage with medium accuracy and resolution. Sparser baselines (the length between neighboring image pairs) do not necessarily generate higher accuracy than those from denser baselines, and an optimal frame network should consider the essential completeness of complex components and potential degeneracy cases. Substituting blur frames with adjacent sharp frames could reduce global errors by 5–15%.

## 1. Introduction

Noncontact measurement methods are widely used in architectural heritage surveys. According to the employed sensors, these methods can be distinguished as range-based modeling via active optical sensors and image-based modeling via passive optical sensors [1]. Presently, one of the most commonly used methods of range-based modeling in architectural heritage is terrestrial laser scanning (TLS). It is capable of measuring 3D geometric data with mm-level accuracy and has been applied to digital documentation of cultural heritage since the 1990s [2]. Image-based modeling developed rapidly in the past decade motivated by structure from motion (SfM) algorithms and imaging sensors. SfM automates the process from tie points extraction to dense points generation without camera pre-calibration [3,4]. It speeds up conventional photogrammetry and facilitates non-expert use. Compact cameras along with external platforms expand the coverage of image acquisition to wider areas such as low-altitude domains [5], historic urban infrastructure [6], and underwater [7]. The progresses in data acquisition promote novel methods of data elaborations from conventional computer-aided drafting (CAD) to building information modeling (BIM) [8] and 3D geographic information system (GIS) [9].

However, in narrow scenes of architectural heritage, data acquisition is not a straightforward task, considering a reasonable budget, portability, and efficiency [10]. Although novel laser scanners (e.g., Leica BLK360) and mobile mapping systems (MMSs) based on simultaneous localization and mapping (SLAM) technology are portable [11,12], they are poor in chromatic data retrieval compared to photogrammetry [13], and limited to high-budget surveys. Photogrammetry is a low-cost and rapid measurement method, but photographing with regular lenses in narrow scenes leads to short baselines (the length between neighboring image pairs), while a large baseline/depth (B/D) ratio is crucial to photogrammetric accuracy [14]. In narrow spaces, the field of view (FOV) of each photograph is limited; hence a large image amount is needed for sufficient overlap. It burdens not only on-site labor intensity but also computation resources in-lab. Compared to classical cameras with regular lenses, spherical cameras offering 360°-FOV can reduce the image amount and avoid excessively short baselines, but they also lead to serious image distortions and nonuniform ground sampling distance (GSD) [15]. Regular lenses follow perspective projections, while fisheye lenses that constitute a spherical camera are designed with different types of projections (e.g., equidistant, equisolid, stereographic, and orthographic), among which equidistance is perhaps the most common [16]:

(1) $$r_{perspective}=\;f\;\theta$$

(2) $$r_{equidistant}=\;f\;\;tan\left(\theta \right)$$

where *θ* is the angle between the principal axis and the incoming ray, *r* is the radial distance from the principal point, and *f* is the focal length.

Studies have been devoted to the calibration of spherical cameras [17]. However, although unit commercial software enables the use of spherical cameras with “one-click operation”, they are not an option for nonexpert users in measurement. In photogrammetry software such as PhotoScan [18], Pix4Dmapper [19], and ContextCapture [20], equirectangular images pre-stitched from multi-lenses are currently supported. Along with low-cost spherical cameras (less than 300 euro), such software packages introduce a novel 3D reconstruction approach for narrow scenes of architectural heritage with high portability, low cost, moderate level of accuracy, and decent chromatic data retrieval [21,22].

When the scales of 3D reconstruction are large, capturing video streams is much more efficient than capturing still images [23]. In narrow scenes of architectural heritage, videographing with a spherical camera while walking not only speeds up on-site work, but also ensures sufficient overlaps between neighboring frames and consequently robust transitions between different scenes (i.e., from exteriors to interiors, from a room to another) [24]. These advantages are helpful to operators without high proficiency and knowledge of estimating image overlap and can be used for rapid measurements in tourist sites. In spite of such conveniences, videogrammetry is not frequently used for architectural heritage surveys focusing on accuracy. In contrast to static images, video frames may suffer from low resolution, blur effects, and redundant overlaps [25]. At present, many low-cost spherical cameras are capable of 3.5 k (ca. 8 million pixel) videography resolution. Larger resolution is expected in the near future with current sensor-upgrade speed. Though such resolution covers 360° FOV, they lead to sufficient GSD in narrow scenes where distances between targets and cameras are usually only a few meters. Hence, we consider here the other two issues—baselines and blur assessment—the main factors impacting the accuracy of spherical-camera videogrammetry.

Frames should be selected before 3D reconstruction, as a full employment is usually unfavorable to accuracy and excessive for computation. Taking 30 frames per second (fps) of the camera and 1 m/s walking speed, the average distances between adjacent frames (baselines) are only 3.3 cm. Even for narrow scenes with limited distances, such baselines are too short to form large B/D ratios. Generally, a shorter B/D ratio gives rise to more tie points, but it also increases reconstruction uncertainties and yields more noise in 3D models. In photogrammetry, a physical point appearing in three images is supposed to be the ideal overlap ratio and the guidance determining optimal camera networks [26]. Using similar principles, several methods select key video frames for 3D reconstruction by achieving a balance between a sufficient overlap and long enough baselines [27]. Improved methods take more factors into consideration, such as reprojection errors, complexity of the scene, and speed of camera movements [28]. In spite of the excellent results these methods might achieve, they do not address the case of spherical cameras. Due to their 360° FOV, overlaps can easily be satisfied from a minimal number of frames with long baselines. In addition, the impact of frame selection on the 3D points accuracy and density of semantic components (floors, walls, reliefs, etc.) given their distinctive geometries, surface contexture, and lighting conditions are rarely addressed, as there was no need to do so from the perspective of computer vision. The work aims at evaluating spherical-camera videogrammetry as a straightforward method for a nonexpert user. Consequently, the frames are simply extracted with different ratios for studying the impact of baselines and integrated with blur assessment of video frames, as discussed in the next paragraph.

At present, many low-cost panoramic cameras have in-built electronic image stabilization. Fundamentally, image stabilization actively removes blur by predicting the future motion from the past one [29]. In addition, external gimbal stabilizers can be used to further reduce camera shake. In addition to hardware methods, several methods have been proposed to handle the problem by modeling motion blur and deblurring them [30], but several minutes are usually required to deblur even a single frame. Given the dense baselines and the fact that only a small proportion of frames are used in our applications, a wise method is simply detecting the most blurred frames and substituting them with sharper adjacent frames. This avoids potential errors in deblurring and intensive computations. One of the most commonly used methods for blur assessment is the Blur Metric [31]. This method evaluates blur effects in the horizontal and vertical directions and reports the results of blur perception with numbers ranging from “0” to “1” (with “0” being the best and “1” the worst). Another convenient blur assessment method is a built-in function in photogrammetry software PhotoScan, named "Estimate Image Quality". It evaluates the sharpness of frames with "black-box" algorithms also by means of the numerical range from zero to higher values (with zero being the worst). These two methods are employed in this study to assess the impact of blur filters on 3D reconstruction accuracy.

**Figure 1:**
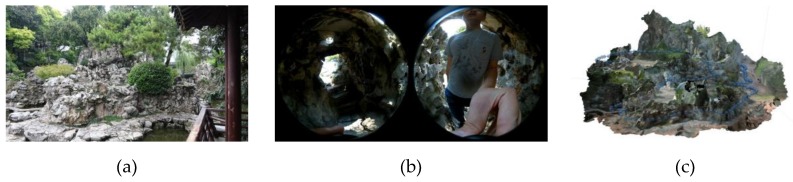
3D reconstruction of the rockery in a Chinese classical garden through combined use of a consumer-level spherical camera and a typical structure from motion (SfM) workflow. **(a)** A view of the north rockery in Zhan Garden, Nanjing; **(b)** an example of raw video frames; and **(c)** the 3D model and recovered frame positions.

In spite of the potential of spherical-camera videogrammetry as a low cost, rapid, and robust measurement method in narrow scenes of architectural heritage, as shown in Figure 1, its application to metric purposes with variable frames extraction ratios and blur filters requires careful study. This work addresses these issues based on two observational studies varying in layouts, surface textures, and lighting conditions. The methodology is simple but effective: first, 3D results are generated from different variables; then, their accuracy is assessed by comparing them with the pre-measured ground truth models (GTMs). The study objective is to provide practical references to nonexpert users videographing with spherical cameras for metric purposes. The relative accuracy, impact of baselines, and impact of blur filters of the tested method, along with the existing 3D reconstruction workflow, could be useful to measure narrow architectural heritage owing to low cost, high portability, and easy operation.

## 2. Materials and Methods

### 2.1. Studied Sites

**Figure 2:**
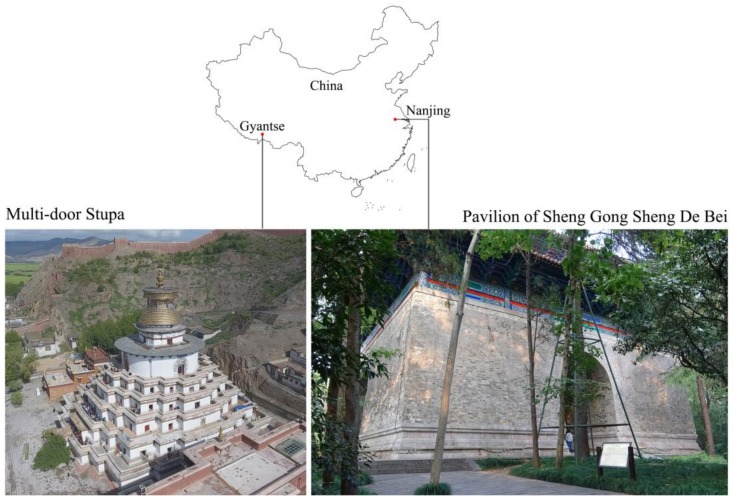
The two surveyed narrow scenes.

Two narrow scenes of architectural heritage with different layouts, surface textures, and lighting conditions are employed for research purposes, as shown in Figure 2. The usage of regular optical measurement methods such as TLS and perspective-camera photogrammetry are limited in both of them. The first scene is a part of the Multi-door Stupa in Gyantse, Tibet, China (denoted as the "Stupa"). Stupas are Buddhist architecture composed of narrowing-upward terraces and chapels. The surveyed scene, the fifth-floor terrace of the Stupa, is an annular corridor defined by terrace enclosures, terrace floors, and walls of the central hall. The dimensions are approximately 67 m in perimeter and 2–3 m in width. TLS on the ground at the site yields an incomplete model without enclosures and floors due to self-occlusions, while aerial images from a UAV covers the enclosures with sharp angles. The second scene is the Pavilion of Shen Gong Sheng De Bei (denoted as the "Pavilion"), the first piece of architecture in the long sequence of the first emperor’s tomb of the Ming Dynasty, located in Nanjing, China. It is also known as "the Square city" for its symmetrical layout and brick-built vaults. As the Pavilion is a part of a property inscribed on the United Nations Educational, Scientific and Cultural Organization (UNESCO) World Heritage List, operations of large measurement devices (i.e., laser scanner, total station) are forbidden on the site. Photogrammetry performed with a perspective camera requires extensive labor for photographing and causes failure-prone transitions between exteriors and interiors.

### 2.2. Video Capture

**Figure 3:**
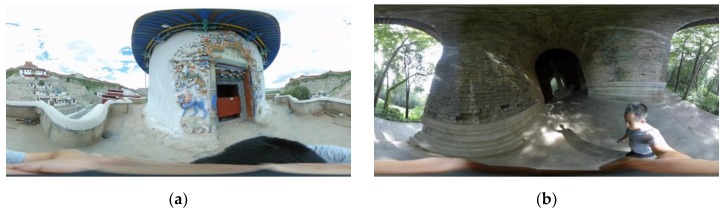
Examples of extracted video frames of the two datasets. (**a**) The Stupa; and (**b**) the Pavilion.

The employed spherical camera was a XiaoMi Mi Sphere (XiaoMi, Beijing, China), as shown in Table 1, owing to its good performance in photogrammetric applications [32]. Equipped with dual 190°-lenses (front-rear), it captures videos at 3.5 K (3840 pixel × 1920 pixel) resolution and 30 fps. It has in-built 6-axis image stabilization for shake reduction. The pocket-size camera can be easily brought to narrow scenes of architectural heritage or strictly managed cultural relic sites. The videos captured by the dual lenses can be automatically stitched in XiaoMi’s own software (Madventure 360) and extracted to equirectangular frames in video editors, as shown in Figure 3.

In the two surveyed sites, videos were captured by lifting the camera above the head of the operator (for reducing the occlusions) while walking at a normal speed along the planned route. The durations of the Stupa dataset and the Pavilion dataset are 1 min 17 s and 3 min 10 s, respectively.

### 2.3. 3D Reconstruction with Variable Baselines and Blur Filters

As equirectangular frames are supposed to be distortion-free, camera pre-calibration is not necessary. The selected frames can be directly fed to photogrammetric software that supports the spherical camera type and turned into a 3D point cloud with full automation. In this study, PhotoScan (version 1.4.4) was used to perform this task with the following parameters:

- Camera type: spherical;
- Align photos: default (accuracy: medium; key point limit: 40,000; tie point limit: 4000);
- Build dense cloud points generation: default (quality: medium; depth filtering: aggressive).

To assess the impact of baselines and blur filters on the achieved accuracy, we carried out 3D reconstruction from predefined groups with the following variables.

#### 2.3.1. Frame Extraction Ratio

The video frames of the Stupa and the Pavilion were extracted into groups denoted with corresponding ratios. Given the stable walking speed, the extraction ratio literally determines the baselines. For example, B_20_ means extracting frames with an interval of 20. Given the 30 fps of the employed device and normal walking speed (approximately 1 m/s), the average baselines of B_20_ were in the range 0.6–0.7 m. Starting from B_10_, the other groups were extracted every 10 frames, i.e., 20, 30, 40, …, n (denoted as B_20_, B_30_, B_40_, …, B_n_), gradually leading to sparser baselines.

#### 2.3.2. Blur Assessment Methods

We detected the most blurred frames and substituted them with sharper adjacent frames extracted from a denser baseline group. To test the impact of blur filters to B_30_, for example, we assessed the blur effect of each frame in B_10_, and enabled only the least blurred frames in every three adjacent ones for 3D reconstruction. This group had the same frame amount and average baselines as the original B_30_. Two blur filters, Blur Metric (denoted as F_bm_) and PhotoScan (denoted as F_ps_) were respectively used for assessment. For both datasets, B_30_ was picked as the tested group.

### 2.4. Accuracy Assessments with GTMs

**Figure 4:**
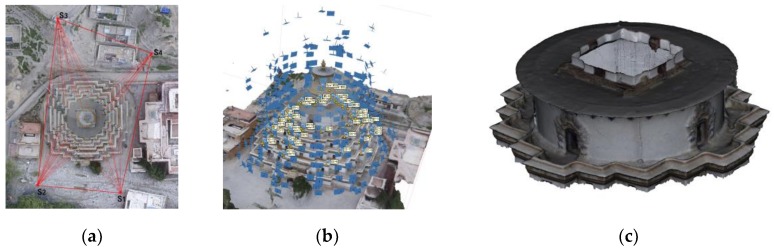
The ground truth model (GTM) of the Stupa. (**a**) The geodetic network measured with a total station; (**b**) image positions and ground control points (GCPs) of the entire Stupa; and (**c**) the mesh model.

**Figure 5:**
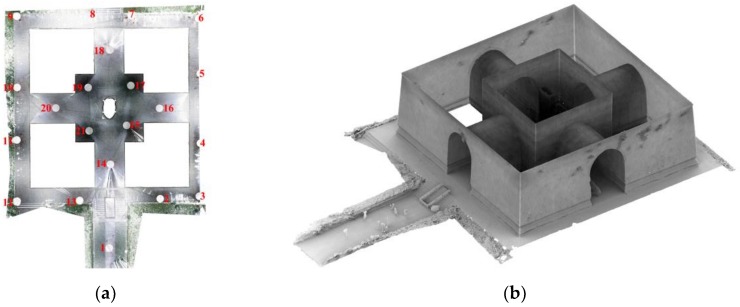
The GTM of the Pavilion. (**a**) The plan and scanning stations (highlighted in red); and (**b**) the mesh model.

Two GTMs were employed respectively to assess the accuracy of each group of the two datasets. The GTM of the Stupa dataset was derived from low-altitude photogrammetry along with 55 ground control points (GCPs) measured from 4 stations with a total station. The GCPs were evenly-distributed natural features (such as the corners of paintings) on the Stupa. More details of the measurement network are available in [5]. Though the photogrammetry-derived model had slightly lower accuracy (RMSE = 2.05 cm, relatively 1/2000 the Stupa’s length) than that derived with a TLS, it ensured a more complete coverage of the terrace due to the oblique aerial images, as shown in Figure 4. The GTM of the Pavilion dataset was derived from a Leica BLK360 laser scanner, as shown in Figure 5. Since the scans were sufficiently overlapped and the Pavilion was feature-abundant, the complete model was automatically registered from 21 stations with mm-level errors (RMSE < 3 mm). The deviations between the tested groups of the two datasets and GTMs were computed via cloud-to-mesh (C2M) distances. Besides GTMs’ own source of errors, the following factors should be considered to ensure the reliability of computation:

- The GTMs and the tested datasets were not exactly corresponding to each other in terms of completeness and density;
- The deviations of the tested groups to the GTMs may not follow a Gaussian distribution.

Consequently, we first subsampled the tested models to 6-mm resolution, which is close to that obtained from TLS, removed the outliers (gross errors deviated more than 1 m) in each group, and finally used the same pairs of natural features and the same parameters (number of iterations: 20; RMS difference: 1 × 10^−5^; sampling limit: 150,000; adjust scale: enable farthest points removal) of the iterative closest points algorithm in CloudCompare [33] to register each model to the GTMs. Considering that the deviations may not follow Gaussian distributions, we analyzed the statistics with not only mean absolute errors and standard deviations but also median absolute errors.

## 3. Results

### 3.1. Impact of Baselines

**Figure 6:**
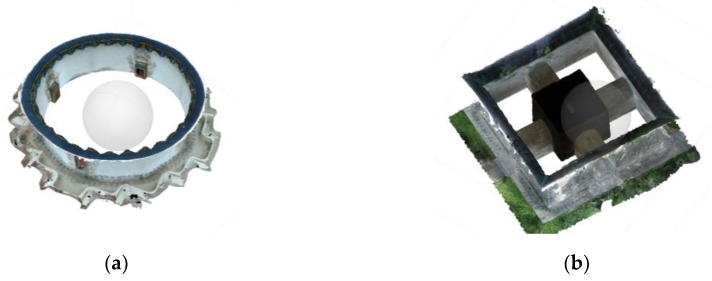
Textured mesh model with recovered frame positions of the two datasets. (**a**) B_30_ of the Stupa; and (**b**) B_30_ of the Pavilion.

In the Stupa, complete frame orientations were achieved from B_10_ to B_40_, and in the Pavilion, from B_10_ to B_50_, as shown in Figure 6. Sparser-baseline groups (i.e., B_50_ of the Stupa and B_60_ of the Pavilion) failed to generate complete 3D models. The RMS reprojection errors grew as the baselines increased except for B_50_ of the Pavilion, as shown in Table 2 and Table 3. Its error was higher than that of B_40_ and close to that of B_30_. RMS reprojection errors are not absolute criteria for assessing accuracy, because they accumulate as the frame amount increases. However, given B_50_ had less frames than B_30_ and B_40_, higher errors suggest that B_50_ was probably less accurate than B_30_ and B_40_ without validation by the GTMs.

**Figure 7:**
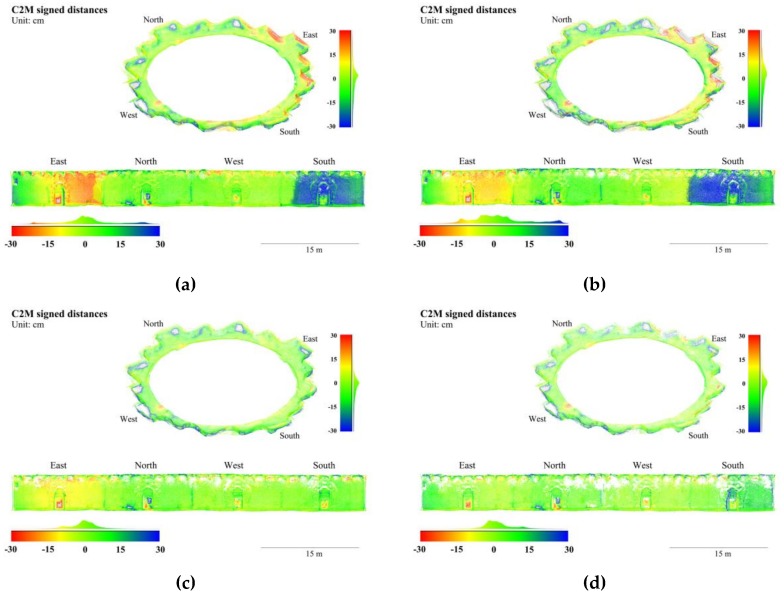
3D points colored in cloud-to-mesh (C2M) signed distances to the GTM with variable baselines of the Stupa. (**a**) B_10_; (**b**) B_20_; (**c**) B_30_; and (**d**) B_40_.

In the Stupa, B_30_ and B_40_ had the smallest mean absolute errors and median absolute errors, respectively, B_20_ had the largest of both, and B_10_ in the middle. The deviations on the wall surfaces of the central hall were more obvious than those on the terrace floor and enclosures, as shown in Figure 7. To visualize the deviations of each group in parallel, the wall surfaces were segmented and unfolded into planar surfaces. Similar deviations were observed in B_10_ and B_20_ in terms of locations and tendencies: the wall near the east portal migrates outwards, and the south portal, inwards. The maximum deviations at these places were greater than ±20 cm in B_10_, as shown in Figure 7a, and greater than ±15 cm in B_20_, as shown in Figure 7b. In B_30_, as shown in Figure 7c, the eastern deviation lightens to a maximum of 10 cm and is not detectable in the southern area. As neither deviations were observed in B_40_, as shown in Figure 7d, we can deduce that, in a frame network with uniform B/D ratios, denser frame baselines tend to increase the distortion effects, given the planar videographed surface and the lack of abundant texture. The deviations of floors were without obvious differences among different groups, while B_30_ and B_40_ had fewer gross errors than those in B_10_ and B_20_ on enclosures. The latter situation was in accordance with their deviations on the wall surfaces.

**Figure 8:**
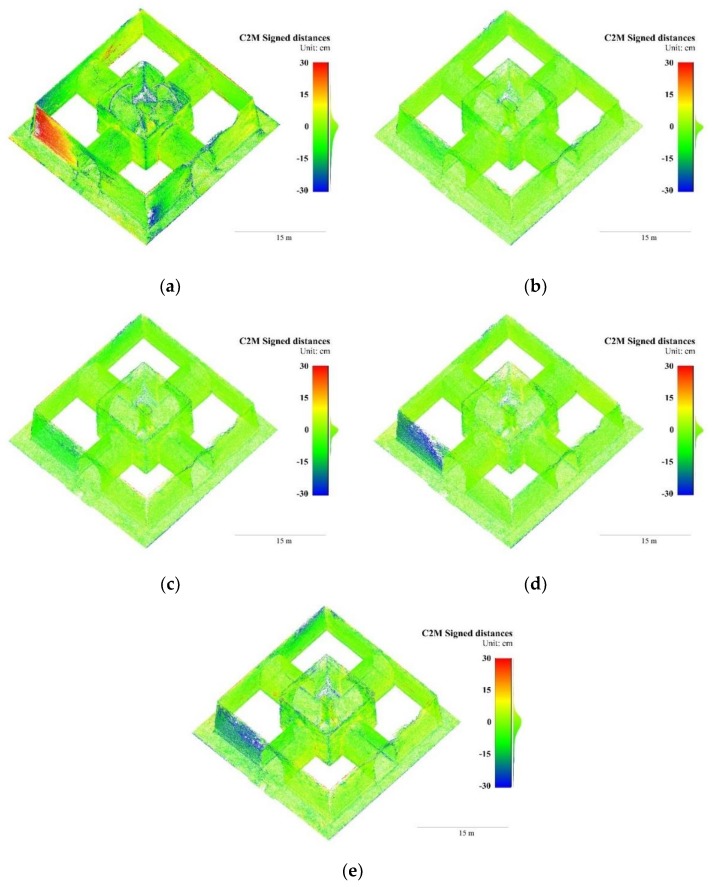
3D points colored in signed distances to the GTM with variable baselines of the Stupa. (**a**) B_10_; (**b**) B_20_; (**c**) B_30_; (**d**) B_40_; and (**e**) B_50_.

In the Pavilion, the best accuracy was achieved in B_20_ and B_30_, followed by B_40_, while B_50_ and B_10_ had the poorest accuracy. The most common deviations in all groups were located on the wall surfaces where two elevations intersect, as shown in Figure 8. The maximum errors in B_10_, as shown in Figure 8a, were close to ±30 cm on the south elevation and the eastern elevation; and greater than 15 cm on the eastern elevation of B_50_, as shown in Figure 8e. These results suggest that, in a complex frame network with variable B/D ratios, excessively dense or sparse baselines may both decrease the accuracy of 3D models and a minimal-amount frame network just satisfying essential overlap does not necessarily lead to the best accuracy. The poor lighting conditions in the Pavilion interiors did not cause gross errors. In all the groups, the interior walls and vaults were reconstructed with satisfactory accuracy.

### 3.2. Impact of Blur Filters

In both datasets, blur filters had positive effects on the accuracy of 3D reconstruction compared with raw frames without any filters, and F_ps_ yielded better results than those with F_bm_.

**Figure 9:**
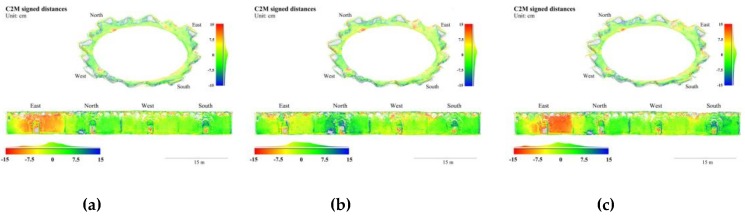
3D points colored in signed distances to the GTM with variable blur filters of the Stupa. (**a**) Raw frames; (**b**) F_ps_; and (**c**) F_bm_.

**Figure 10:**
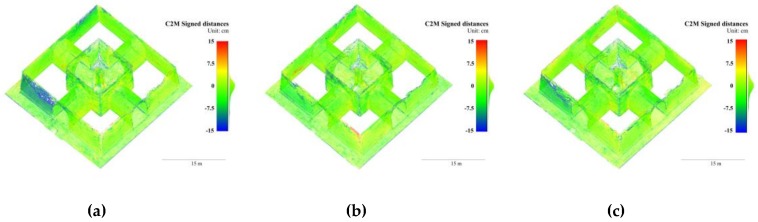
3D points colored in signed distances to the GTM with variable blur filters of the Pavilion. (**a**) Raw frames; (**b**) F_ps_; and (**c**) F_bm_.

Compared with raw frames, the median absolute errors and mean absolute errors in F_ps_ reduced 15% and 5% in the Stupa, respectively, and 7% and 12% in the Pavilion, as shown in Table 4 and Table 5. The range of color-coded deviations were shortened to ±15 cm to reveal the subtle changes. In the Stupa, the distorted eastern wall surfaces generated from raw frames were much less apparent in those from F_ps_, as shown in Figure 9a,b, while in the Pavilion, the deviations on the south elevation were also apparently reduced, as shown in Figure 10a,b. The standard deviations of F_ps_ were 4% and 27% less than raw frames in the Stupa and Pavilion. Given the Stupa is an exterior and the Pavilion is mainly interior, the filters had more impact on the scenes with poor lighting conditions.

Though RMS reprojection errors of F_bm_ were much smaller than the raw frames in both datasets, the statistics and color-coded deviations were not fully consistent with this trend, as shown in Table 4 and Table 5. Compared with raw frames, the impacts of F_bm_ were not obvious in both datasets in terms of the median absolute errors and the mean absolute errors. The reduced color-coded deviations in the Pavilion was notable, as shown in Figure 10a,c, as the blue areas were lightened, but ambiguous in the Stupa, as shown in Figure 9a,c.

## 4. Discussion

### 4.1. Potential Applications

The field of architectural heritage has been experiencing advancements in 3D measurement technologies resulting from developments in optical sensors and computer vision algorithms in the past few years. However, generating complete, accurate, and sufficiently dense 3D models for BIM and 3D-GIS with high levels of detail is difficult. The use of optical measurement technologies is largely dependent on the geometries of the measured objects. Surfaces exposed to ample spaces (e.g., facades of churches, palazzos, and villas) are ideal for measurement with TLS or photogrammetry. When the surfaces are exposed to narrow spaces, TLS may prove to be too labor-intensive on-site and susceptible to error-prone data registrations. Further, the resultant divergent camera network may even invalidate the photogrammetry process if the baselines are excessively short. Such situations are common worldwide (e.g., corridors, staircases, and tunnels), and unique types exist from region to region, such as urban porticos in Europe and classical gardens in China and Japan. Photogrammetry with a handheld camera has been proven to be an effective method of generating accurate 3D models in the case of porticos in Bologna, Italy [6], but on-site efficiency hinders its application to the complete 42-km portico of the city, as a 20-m fragment would require more than 200 images and require a few hours. Similarly, integrated use of TLS, terrestrial photogrammetry, and UAV photogrammetry is essential to yield complete 3D models of Chinese classical gardens, but the intensiveness of the required labor is high, especially for the rockeries [34]. Spherical-camera videogrammetry is a promising method for solving such problems, as shown in Figure 1. This study was conducted to investigate its metric accuracy based on two case studies.

**Figure 11:**
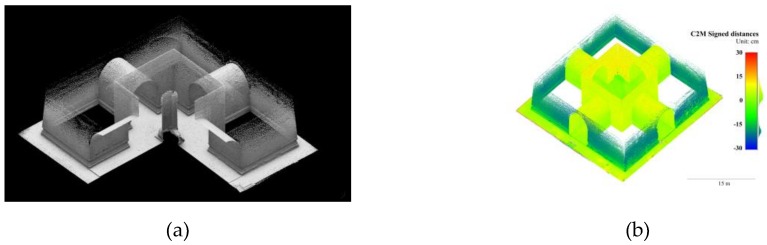
3D model of the Pavilion dataset obtained with mobile mapping system (MMS; GeoSLAM ZEB-REVO handheld scanner). Compared with that from spherical-camera videogrammetry, the MMS-derived model is **(a)** less-noisy and **(b)** has similar global accuracy, but has obvious disjunctions between the outer walls and the interiors.

The accuracy achieved with the XiaoMi Mi Sphere Camera along with commercial photogrammetry software is in the range 0.5–1.5 pixels according to [32], but this result was obtained based on photographing a planar wall surface with static images from the front lens, as opposed to hundreds of equirectangular video frames stitched from dual lenses recording complex scenes. In our studies, the relative errors in the five groups ranged from 1/700 in B_10_ to 1/2000 in B_20_, given the dimensions of the Pavilion (26.5 × 26.5 × 9.2 m). Because of the varying site situations, such as the stacks of restoration materials, the relative errors in the Stupa were greater than those of the Pavilion, but they are still below 1/500 in the worst case (B_20_). Although such a level of accuracy is not comparable to those achieved with TLS, it is close to those achieved with MMS, as shown in Figure 11, (absolute mean error = 3.85 cm and standard deviation = 3.33 cm). This MMS-derived model was generated with a GeoSLAM ZEB-REVO handheld scanner (GeoSLAM, Ruddington, UK) while walking in a similar way (speed and route) with videographing. Though the scanning takes only 5 minutes, it lacks color and texture and suffers from serious reduction of point density due to limited scanning radius, as shown in Figure 11. The resulting completeness and accuracy of spherical videogrammetry caters to medium-scale CAD and historic building information modeling (HBIM), but is not sufficient for as-built BIM that aims at documenting the imperfect situations of architectural heritage [35]. It is worth noting that such accuracy levels are achieved without integrating GCPs. In general, GCP integration can refine the recovered image/frame orientations in 3D reconstruction via bundle adjustment [36]. Hence, better spherical videogrammetry accuracy could be expected with accurate and evenly distributed GCPs measured with a total station.

**Figure 12:**
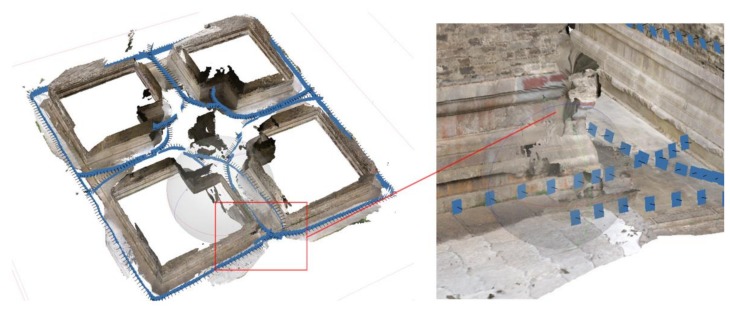
3D model of the Pavilion dataset obtained with perspective-camera videogrammetry. The model of the pedestals is obviously warped.

**Figure 13:**
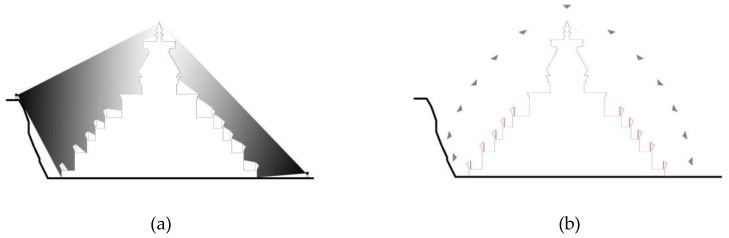
Coverage of TLS (**a**) and UAV-photogrammetry (**b**) in the Stupa dataset. Although the latter method generates a more complete model than that from TLS, the inner surfaces of the enclosures on each floor (highlighted in red) are still missing considering reasonable labor intensity and favorable camera network.

Owing to extremely wide FOV and manageable frame extraction ratios, the frame alignments of spherical cameras are robust to scene transitions, lighting alternations, and poor texture. Perspective cameras, however, are error-prone in such conditions, as shown in Figure 12. In addition, spherical-camera videogrammetry has promising on-site efficiency compared to other optical measurement methods. Only a few minutes is spent videographing the two scenes, whereas measuring the same objects with TLS or perspective-camera photogrammetry would require far more time, as shown in Table 6, more money, and pose device portability problems. It could provide supplementary measurement for areas where using TLS and perspective-camera photogrammetry is inefficient, as shown in Figure 13.

In summary, the accuracy, efficiency, and portability of spherical photogrammetry make it a promising measurement method for 3D reconstruction of narrow architectural heritage such as staircases, long corridors, and rockeries in Chinese classical gardens.

### 4.2. Results Analysis and Future Developments

**Figure 14:**
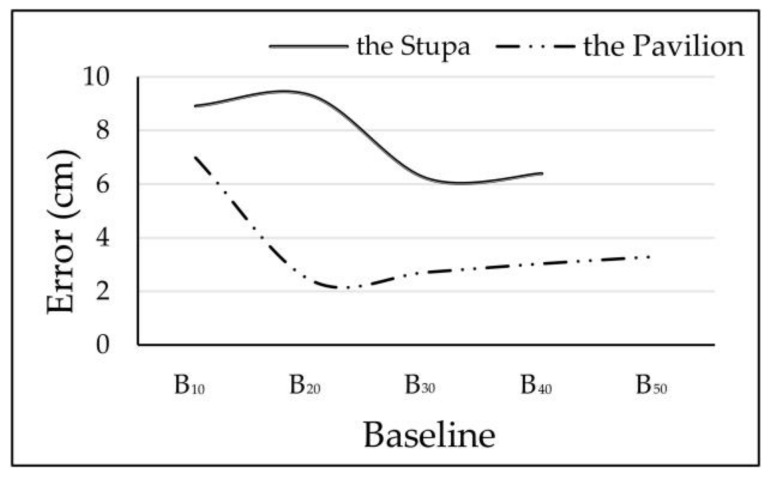
Median absolute errors with variable baselines in the two datasets.

In terms of the median absolute errors, the best accuracy in the Stupa was achieved from the group with the largest baselines (B_40_), while in the Pavilion, accuracy decreased from B_20_ and B_50_ as baselines become larger, as shown in Figure 14. In both datasets, consistent correlations between increasing baselines and declining accuracy were not observed. We can further deduce that more criteria should be considered along with baselines for extracting optimal frames if they are produced from a spherical camera.

**Figure 15:**
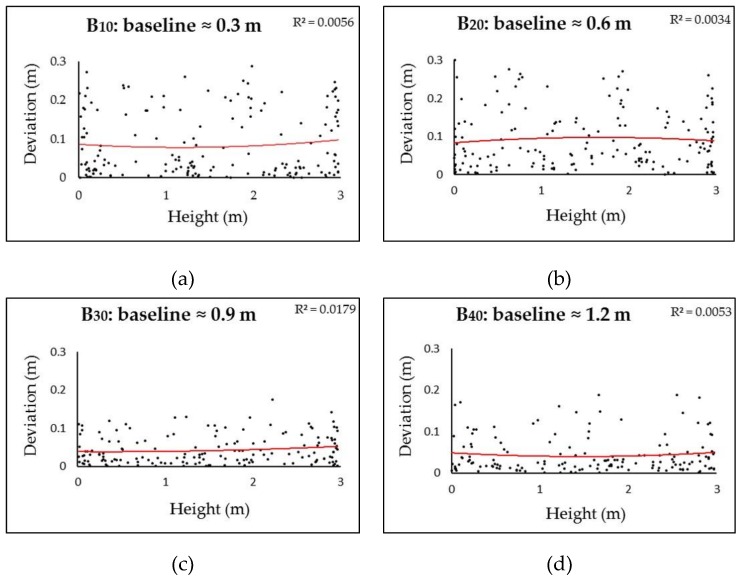
Correlations between height (vertical distance to the base plane) and deviations (to the GTM) in the Stupa dataset with variable baselines. Two-polynomial trend lines (in red) with correlation coefficient (R^2^) are given.

**Figure 16:**
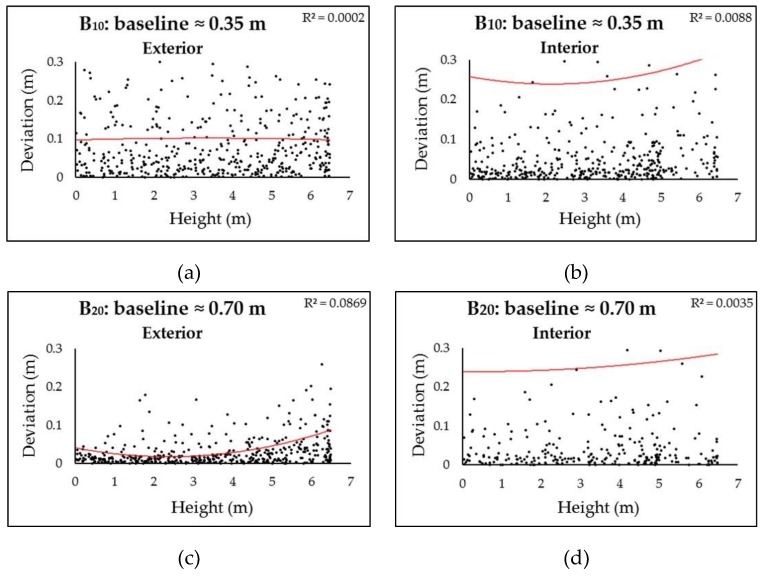

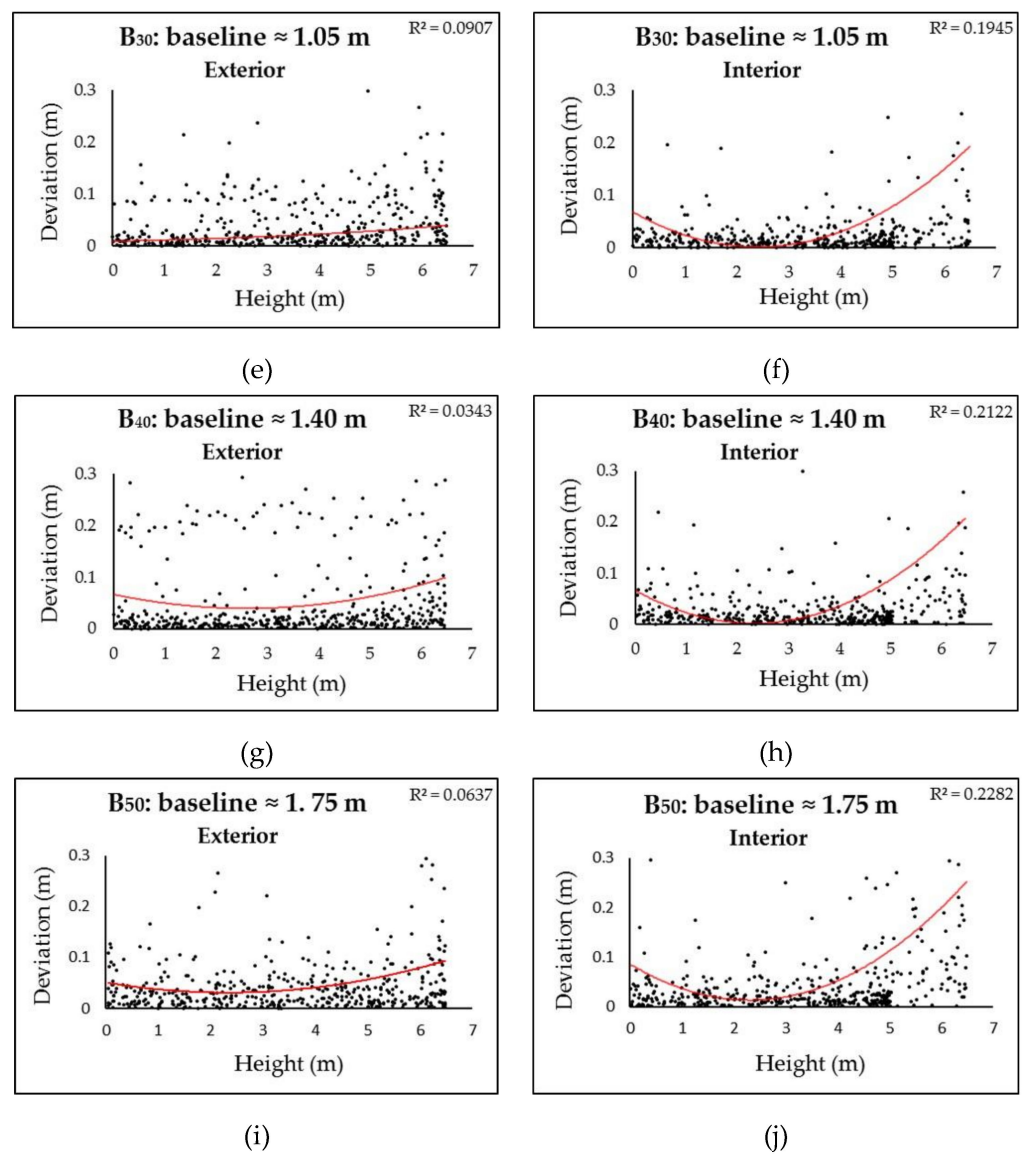
Correlations between height (vertical distance to the base plane) and deviations (to the GTM) in the Pavilion dataset with variable baselines. Two-polynomial trend lines (in red) with correlation coefficient (R^2^) are given. (**a**) (**c**) (**e**) (**g**) (**i**) represent exterior walls; and (**b**) (**d**) (**f**) (**h**) (**j**) represent interior walls and vaults.

**Figure 17:**
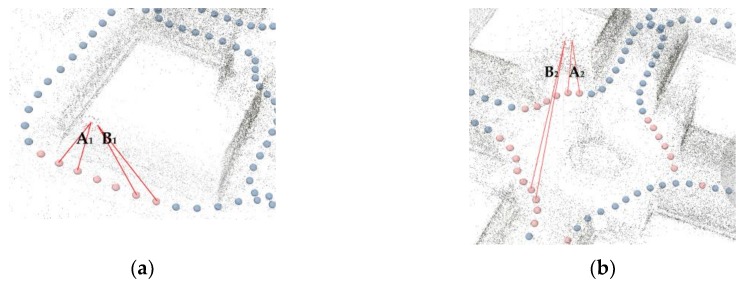
Triangulations between tie points and corresponding frames in the Pavilion. (**a**) A part near the top of the exterior walls; and (**b**) a part near the top of the interior walls. While their maximum intersection angles are similar (A_1_ ≈ A_2_), their minimums are very different (B1 > B2).

A unique feature of spherical cameras, in contrast to perspective cameras, is that the resultant GSDs seriously degrade from frame center to marginal areas. Given the same baseline, the 3D points in the physical spaces imaged close to the frame epipoles suffer from the sharper intersection angles of the two homologue frame rays, and consequently, higher localization uncertainties than those close to the principal point. Earlier studies discarded the marginal areas and used only the central contents for 3D reconstruction [15]. We took a different approach in order to assess the impact of radial distortion on the accuracy. We defined the height of the camera positions as the base plane, and analyzed the correlations between the relative height of 3D points (subsampled to 1 point per m^2^) and their accuracy (deviations to the GTMs). The analysis of the Stupa involved only the walls of the central hall. The Pavilion was segmented into two areas: the exterior parts (walls) videographed with uniform B/D ratios, and the interior parts (walls and vaults) with variable and complex B/D ratios. In both datasets, consistent linear correlations between increases in height and decreases in accuracy were not observed, as shown in Figure 15 and Figure 16. However, it was noticeable that (1) B_10_ and B_20_ in both datasets yielded very noisy point clouds regardless of variations in height, which suggests that baselines less than 1 m should not be employed in the studied cases; and (2) in the Pavilion, the negative impact of height on accuracy was much more obvious than that in the exterior, which is perhaps related to their different frame network geometries, as shown in Figure 17. Although they appeared noisy, the interior surfaces did not suffer from distortions (±15 cm) as obvious as those on the exterior surfaces in both datasets, as shown in Figure 7a,b,c, and Figure 8a. These failures can perhaps be ascribed to degeneracy cases: the 3D points in the physical spaces are coplanar, and corresponding camera motions are homogeneous without sufficient translations and rotations [37]. Such observations suggest that different strategies should be employed for façades and interiors. Future studies will test whether an irregular walking route and active camera movements along with existing software methods for regular-lens frames [38] could reduce degeneracy cases for façades, and frame amount reduction, content cropping (discarding the seriously distorted marginal areas), and tie points constraints could reduce noise in interiors.

Although this work focused on the accuracy of 3D models, achieving a higher accuracy should always be considered along with sufficient completeness in practice. In our tests, the concise integrity of the Pavilion was ascribed to the exclusive use of bricks, and the impact of baselines on the completeness of semantic components was not obvious. In spite of a lower resolution than B_10_, B_50_ yielded a model without apparent blank areas. However, the Stupa is composed of not only walls, but also Tou-kungs, the painted timber brackets supporting the overhung roofs on top of the walls. Three-dimensional reconstruction of Tou-kungs requires much denser frame overlaps than walls. As a result, the holes on Tou-kungs accumulated rapidly as baselines became sparser, as shown in Figure 7. B_10_ and B_40_ yielded point clouds with similar resolution on walls, but dramatically different on Tou-kungs. Although B_40_ yielded the optimal accuracy globally, B_10_ was a better baseline for mapping Tou-kungs in spite of lower accuracy. To achieve a balance between accuracy and completeness on different semantic components, future studies will mainly be conducted in the frame network: adding a sequence of denser baseline frames on higher levels than the current one by mounting the cameras closer to the Tou-kungs. It is theorized that two frame sequences will reduce the above-mentioned degeneracy cases.

Filtering of blur frames has been proven to be an effective quality control method in tests and is suggested for application. However, because the filter in PhotoScan is a “black box”, open source algorithms are expected to be used in the future to test their impact on accuracy with variable surface textures, illumination, and frame amounts. In the photogrammetric pipeline, preprocessing images such as color calibration and content enhancement can increase the accuracy level, while raw images without compression are needed [39]. Currently, high-end video cameras can output 4 K resolution-raw format video, but low-cost spherical cameras output only compressed video formats (e.g., MPEG-4) and, consequently, the frame format is in .jpg. An extended quality control pipeline for frames from blur detection to the existing methods on raw-format images is expected in future studies.

In this study, 3D reconstruction was based on the commercial software PhotoScan. It is worth noting that the results of 3D reconstruction are not deterministic given the same frames in different software or even in the same software. This may be due to the use of random seeds (e.g., random sample consensus: RANSAC [40]) as open source SfM software do [41], but only limited information about the used algorithms of commercial software is available. This may affect the precision of accuracy validation in this study, but the impact is slight, as shown in similar studies in terms of using PhotoScan for evaluation purposes [42]. The use of open source software in the future would facilitate more interventions and feedback during 3D reconstruction.

## 5. Conclusions

This study evaluated the accuracy of 3D reconstruction using video frames from low-cost spherical cameras via two architectural heritage case studies. This was performed with the objective of assessing whether it is a valid measurement method for narrow scenes, and observing how baselines and blur filters affect the results. The following conclusions are drawn:

- Videogrammetry with consumer-level spherical cameras is a robust method for surveying narrow architectural heritage, where the use of other optical measurement technologies (e.g., TLS, MMS, and perspective-camera photogrammetry/videogrammetry) is limited. The wide FOV and manageable frame extraction ratios lead to frame alignments robust to scene and lighting variations. It is low-cost, portable, fast, and easy to use for even nonexpert users.
- The achieved metric accuracy is at cm levels, relatively 1/500–1/2000 in both datasets of our tests. Although it is not comparable to those achieved with TLS or photogrammetry (coupled with precise GCPs and image processing), it is close to that achieved with MMS, and caters to surveying and mapping with medium accuracy and resolution in short periods. Such levels of accuracy, along with low-cost and portability, make it a promising method for surveying narrow architectural heritage in extreme conditions, such as remote areas.
- Baselines and blur filters are crucial factors to the accuracy of 3D reconstruction. Consistent correlations between baselines and accuracy, as those for perspective camera, were not observed in the tests. Relatively short baselines (<1 m) yield point clouds with more noise, but larger baselines do not necessarily lead to higher accuracy. An optimal frame extraction for videos from spherical cameras should consider radial distortions, degeneracy cases, and essential point density. Both blur filters had a positive impact on the accuracy in the tests: substituting blur frames with adjacent sharp frames can reduce global errors by 5–15%.
- Future developments will involve testing of different strategies for façades and for interiors, more layouts of architectural heritage, video processing algorithms, and emerging imaging sensors.

## Figures and Tables

**Table 1:** Specifications of the employed camera.

Dimensions	7.8 × 6.8 × 2.4 cm
Weight (batteries included)	108 g
Lens	f/2.0
Sensor size	1/2.3 inch (6.17 × 4.55 mm)
Photography resolution	6912 × 3456 pixels
Videography resolution	2304 × 1152 pixels, 60 fps; 3840 × 1920 pixels, 30 fps
Format	Photo: DNG, JPG; Video: MPEG-4, H.264

**Table 2:** Statistic results of the accuracy assessment of the Stupa with variable baselines. In the brackets of the first line are the number of frames.

	B_10_(222)	B_20_(111)	B_30_(74)	B_40_(56)
RMS reprojection error (pixel)	1.30	1.14	1.12	1.03
Standard deviation (cm)	±12.23	±12.26	±9.44	±9.20
Mean absolute error (cm)	8.91	9.30	6.21	6.39
Median absolute error (cm)	5.70	6.41	4.38	3.70

**Table 3:** Statistic results of the accuracy assessment of the Pavilion with variable baselines. In the brackets of the first line are the number of frames.

	B_10_(571)	B_20_(286)	B_30_(191)	B_40_(143)	B_50_(115)
RMS reprojection error (pixel)	2.25	1.62	1.54	1.41	1.53
Standard deviation (cm)	±10.26	±4.29	±5.53	±5.58	±5.02
Mean absolute error (cm)	6.98	2.41	2.71	3.03	3.30
Median absolute error (cm)	3.93	1.38	1.53	1.53	2.11

**Table 4:** Statistic results of the accuracy assessment of the Stupa: raw frames and blur-filtered frames.

	Raw	F_ps_	F_bm_
RMS reprojection error (pixel)	1.12	0.95	0.94
Standard deviation (cm)	±9.44	±9.09	±10.15
Mean absolute error (cm)	6.21	6.06	6.90
Median absolute error (cm)	4.38	3.74	4.38

**Table 5:** Statistic results of the accuracy assessment of the Pavilion: raw frames and blur-filtered frames.

	Raw	F_ps_	F_bm_
RMS reprojection error (pixel)	1.54	1.34	1.41
Standard deviation (cm)	±5.53	±4.06	±4.35
Mean absolute error (cm)	2.71	2.39	2.54
Median absolute error (cm)	1.53	1.42	1.53

**Table 6:** Comparison of on-site time of the tested method with other optical measurement methods. TLS: terrestrial laser scanning.

	Stupa (min)	Pavilion (min)
Spherical-camera videogrammetry	2	4
TLS (i.e., Leica BLK 360)	ca. 60	120
Perspective-camera photogrammetry	ca. 60–120	ca. 100–200
